# Pan-genome dynamics of *Pseudomonas* gene complements enriched across hexachlorocyclohexane dumpsite

**DOI:** 10.1186/s12864-015-1488-2

**Published:** 2015-04-18

**Authors:** Anukriti Sharma, Naseer Sangwan, Vivek Negi, Puneet Kohli, Jitendra Paul Khurana, Desiraju Lakshmi Narsimha Rao, Rup Lal

**Affiliations:** Department of Zoology, University of Delhi, New Delhi, 110007 India; Interdisciplinary Centre for Plant Genomics & Department of Plant Molecular Biology, University of Delhi South Campus, New Delhi, India; All India Network Project on Soil Biodiversity and Biofertilizers, Indian Institute of Soil Science, Bhopal, 462038 India

**Keywords:** *Pseudomonas*, Integron, Comparative genomics, Metagenomics, Horizontal gene transfer, Selective pressures

## Abstract

**Background:**

Phylogenetic heterogeneity across *Pseudomonas* genus is complemented by its diverse genome architecture enriched by accessory genetic elements (plasmids, transposons, and integrons) conferring resistance across this genus. Here, we sequenced a stress tolerant genotype i.e. *Pseudomonas* sp. strain RL isolated from a hexachlorocyclohexane (HCH) contaminated pond (45 mg of total HCH g^−1^ sediment) and further compared its gene repertoire with 17 reference ecotypes belonging to *P. stutzeri*, *P. mendocina*, *P. aeruginosa*, *P. psychrotolerans* and *P. denitrificans,* representing metabolically diverse ecosystems (i.e. marine, clinical, and soil/sludge). Metagenomic data from HCH contaminated pond sediment and similar HCH contaminated sites were further used to analyze the pan-genome dynamics of *Pseudomonas* genotypes enriched across increasing HCH gradient.

**Results:**

Although strain RL demonstrated clear species demarcation (ANI ≤ 80.03%) from the rest of its phylogenetic relatives, it was found to be closest to *P. stutzeri* clade which was further complemented functionally. Comparative functional analysis elucidated strain specific enrichment of metabolic pathways like α-linoleic acid degradation and carbazole degradation in *Pseudomonas* sp. strain RL and *P. stutzeri* XLDN-R, respectively. Composition based methods (%codon bias and %G + C difference) further highlighted the significance of horizontal gene transfer (HGT) in evolution of nitrogen metabolism, two-component system (TCS) and methionine metabolism across the *Pseudomonas* genomes used in this study. An intact mobile class-I integron (3,552 bp) with a captured gene cassette encoding for dihydrofolate reductase (*dhfra1*) was detected in strain RL, distinctly demarcated from other integron harboring species (i.e. *P. aeruginosa*, *P. stutzeri,* and *P. putida*). Mobility of this integron was confirmed by its association with Tnp21-like transposon (95% identity) suggesting stress specific mobilization across HCH contaminated sites. Metagenomics data from pond sediment and recently surveyed HCH adulterated soils revealed the *in situ* enrichment of integron associated transposase gene (TnpA6100) across increasing HCH contamination (0.7 to 450 mg HCH g^−1^ of soil).

**Conclusions:**

Unlocking the potential of comparative genomics supplemented with metagenomics, we have attempted to resolve the environment and strain specific demarcations across 18 *Pseudomonas* gene complements. Pan-genome analyses of these strains indicate at astoundingly diverse metabolic strategies and provide genetic basis for the cosmopolitan existence of this taxon.

**Electronic supplementary material:**

The online version of this article (doi:10.1186/s12864-015-1488-2) contains supplementary material, which is available to authorized users.

## Background

*Pseudomonas* represents ubiquitous taxon defined by Gram negative, rod-shaped -γ *Proteobacteria*, having an enormous metabolic versatility to inhabit varied stressed environments [1]. Till date, genus *Pseudomonas* encompasses 218 authentically pronounced species [2] including various niche-specialist genotypes e.g. *P. aeruginosa* [3], *P. stutzeri* [4], and *P. putida* [5]. *Pseudomonas* genotypes are characterized by extensive genetic heterogeneity acted upon by selective pressures through mobile genetic elements (MGEs) that confer catabolic potential to degrade variety of xenobiotic compounds such as antibiotics, biocides, and heavy metals [6].

Over the last two decades, comparative genomics has emerged as a powerful tool to demarcate between functionally important genetic elements (i.e. core-genome) and niche specific adaptive genes on genomic islands (i.e. flexible-genome) [7]. However, impedance to the comparative genomics approach is the inability to demonstrate results at community (population) level which can be circumvented using metagenome data to reveal complex community interactions. Genome size variations among pseudomonads (ranging from 3.7 Mbp for *P. stutzeri* [4] to 7.1 Mbp for *P. aeruginosa* [3]) and their relatively higher *in situ* abundance across stressed environments [8] make them a potential candidate for studying micro-evolutionary events that occur at population level.

Microbial biogeography of HCH contaminated environments has been elucidated using both culture-dependent [9-15] and culture-independent [8,16] studies. Recent metagenomic surveys have revealed that higher HCH contamination (450 mg HCH g^−1^) results in enrichment of sphingomonads and pseudomonads precisely [8]. Competence among sphingomonads to tolerate and/or degrade HCH has been attributed to the acquisition of *lin* genes and studied in details in the past [17-21]. But the genetic mechanism behind co-existence of pseudomonads under HCH stress is still unknown. Here, we have sequenced a HCH tolerant *Pseudomonas* strain i.e. *Pseudomonas* sp. strain RL isolated from HCH contaminated pond sediment. Detailed comparative genomic analysis with its reference ecotypes (n = 17) combined with *in situ* population level genetic information (metagenomics), was performed to highlight the niche specific gene repertoire of strain RL. We further reported a mobile class-I integron (3,552 bp) in strain RL with a captured gene cassette encoding for dihydrofolate reductase (*dhfra1*) gene, associated with a Tnp21-like transposon. Integrons though have been attributed to the acquisition of antibiotic resistant genes since 1980’s but till date their occurrence in *Pseudomonas* spp. has been rare and precisely limited to *P. aeruginosa*, *P. putida*, and *P. stutzeri* [22]. Environmental sequence data from the HCH contaminated pond sediment (45 mg HCH g^−1^) together with previous metagenomic survey of HCH contaminated dumpsite (450 mg HCH g^−1^) and adjoining agricultural soils (0.07 mg HCH g^−1^) [8] were also used to reveal the metagenomic recruitment of this integron unit. Our results highlighted the *in situ* genetic predominance of the integron associated TnpA6100 gene (IS*6100* related transposase) across increasing HCH contamination. Remarkably higher *in situ* abundance of this transposase particularly at the HCH contaminated site emphasizes at the transposon driven HGT among *in situ* cohorts, hence contributing towards the overall fitness of the inhabiting *Pseudomonas* community [23].

## Methods

### Site selection and physiochemical analysis

Field experiment was performed in September of 2011 at HCH contaminated pond located in the vicinity of lindane production unit (28°54′ N, 81° 09′ E) at Chinhat, Lucknow, India. Four random sediment samples, 50 g each were collected from the selected site, transported on ice (4 °C) and stored at −80°C until further processing. Physicochemical analysis of the samples was performed using X-Ray diffraction (XRD) [24], followed by estimation of HCH concentration using previously described method [25]. Briefly, 1 g of each sample was extracted in solvent (hexane/acetone; 1:1) by sonication and the extract was dried to subsequently yield HCH residues. The dried extract was then dissolved in 2 ml ethyl acetate. Further, the concentration of HCH isomers was estimated based on calibration against external standards of HCH isomers (α-, β-, γ-, δ- HCH) by Gas Chromatograph (GC-17A, Shimadzu, Japan) equipped with electron capture ^63^Ni detector and a 30 m REX-50 column (Restek, USA). Gas chromatography data for HCH concentration from another HCH dumpsite survey [8] was also used for comparative evaluation.

### Strain isolation and genome sequencing

Sediment sample (1 g) from HCH contaminated pond was suspended in 5 ml sterile 1× PBS (137 mM NaCl, 1.3 mM KCl, 3.2 mM Na_2_HPO_4_, 0.5 mM KH_2_PO_4_, pH 7.4), serially diluted and plated on Luria-Bertani (LB) agar plates with Nystatin (100 U/ml) [26]. Yellow pigmented colonies were avoided with an aim to isolate *Pseudomonas* strains and not the previously well characterized sphingomonads [9-15,27]. A black pigment producing colony, within 9 days of incubation at 28°C, was picked and purified by repeated streaking on LB agar. Purified bacterial colony was cultured in LB broth; bacterial cells were harvested by brief centrifugations followed by isolation of genomic DNA using CTAB method [28]. Identification of isolated *Pseudomonas* sp. strain RL was confirmed by 16S rRNA gene amplification and sequencing. Total genomic DNA samples were further processed for whole genome sequencing using DNA sample preparation kit (Illumina Inc., San Diego, CA, USA). Paired-end reads were generated using Illumina HiSeq 2000 (n = 6,255,556, 2 kb paired-end library) and 454 GS FLX titanium platforms (n = 1,01,139, 2 kb single-read library) at Beijing Genome Institute, BGI, Shenzhen, Guangdong, China. Whole genome reads were quality filtered using quality measures such as minimum quality score = Q_20_, minimum read length = 90 bp (Illumina) and 350 bp (Pyrosequencing) without ambiguous bases.

### *de-novo* genome assembly and annotation

Raw sequence reads obtained for strain RL were assembled into contigs using Velvet_1.2.03 assembler [29] set at parameters: insert length = 2 kb, standard deviation of insert length = 100 bp, expected coverage = 20 and minimum contig length = 500 bp. After finalizing the *de-novo* assembly, contigs were checked for the misassembled and low coverage regions. Quality filtered contigs were further extended using paired-end criterion based method. Final assembly was checked for the percentage completeness using single copy genes i.e. thirty-one protein encoding phylogenetic marker genes (*dnaG*, *frr*, *infC*, *nusA*, *pgk*, *pyrG*, *rplA*, *rplB*, *rplC*, *rplD*, *rplE*, *rplF*, *rplK*, *rplL*, *rplM*, *rplN*, *rplP*, *rplS*, *rplT*, *rpmA*, *rpoB*, *rpsB*, *rpsC*, *rpsE*, *rpsI*, *rpsJ*, *rpsK*, *rpsM*, *rpsS*, *smpB*, and *tsf)* [30] and 107 genes by Dupont *et al*., (2012) [31]. The genome revealed presence of all the 31/31 and 103/107 genes suggesting its near complete status for further whole genome based analysis. ORFs were predicted from contigs using FragGeneScan 1.16 [32] followed by KAAS (KEGG Automatic Annotation Server) assignment of KEGG ortholog (KO) identifiers to the query genes by BLASTP against the KEGG GENES database [33]. For comparative genome analysis, sequence data was used for *Pseudomonas* genotypes (n = 18, including strain RL) from NCBI (ftp://ftp.ncbi.nlm.nih.gov/genomes/Bacteria/). Summary of the genomes used in this study and their selection criterion is provided in a separate section below under the title ‘Summary of reference genomes and metagenomes’. Metabolic pathways were reconstructed and filtered using MinPath (Minimal set of Pathways) [34] for all the *Pseudomonas* genomes i.e. *Pseudomonas* sp. strain RL and its 17 reference genotypes. Protein family reconstruction was estimated against Pfam [35] and KEGG databases [36] using HMMER [37] and BLASTP, respectively. The strains were further investigated for presence of integron by BLASTN against INTEGRALL 1.2 database [38]. This was followed by a local BLASTN search against a manually curated database pertaining to class-I integrons from *Pseudomonas* genus [38].

### Phylogenomic analysis

Phylogenetic status of strain RL was determined using 400 conserved bacterial marker genes [39] and representative whole genome sequences from 40 diverse *Pseudomonas* species as available in NCBI database (http://www.ncbi.nlm.nih.gov/genome/browse/representative/). In addition to using one representative strain from each of 40 species, 10 supplementary strains (sub-species) from *P. aeruginosa* (n = 4), *P. stutzeri* (n = 4), and *P. mendocina* (n = 2) were also included in the dataset for being assigned as exclusive 10 closest phylogenetic neighbors by RAST whole genome based functional score [40]. A phylogenetic tree was hence constructed using maximum likelihood (ML) methodology on the basis of protein coding bacterial conserved gene (400) sequences [39] determined for *Pseudomonas* sp. RL and 50 other *Pseudomonas* strains with *Azotobacter vinelandii* being the outgroup. *Azotobacter vinelandii* CA was used as outgroup considering its adjacency with pseudomonads (being the sister taxon) but definitely an outlier for the in-group as an independent genus [41,42]. For further studying the sub-species demarcation patterns, pairwise average nucleotide identity (ANI) [43] calculations were performed for explicitly strain RL and its 17 closely related reference genotypes as observed from the clade topology of 400 genes based phylogenetic tree. These 18 *Pseudomonas* strains were then used for further comprehensive comparative genomic analysis.

### Identification of orthologous segments and detection of positively selected proteins

Orthologous segments were determined using Murasaki [44] and OSfinder v1.4 (Orthologous-Segment Finder) [45] for all 18 *Pseudomonas* genomes. Short homologous regions called anchors were determined between the genomes using Murasaki at 28 and 36, weight and length of seed patterns, respectively. Positions of the anchors (as determined by Murasaki) were fed to OSfinder (1000 minimum orthologous segment length cut-off) that discriminated orthologous anchors from non-orthologous anchors using Hidden Markov Model (HMM). To further supplement the above analysis pairwise orthologous gene identification was performed using reciprocal smallest distance (RSD) algorithm [46] set at E-value and divergence cut-off of 1e-15 and 0.5, respectively. Using whole genome based synteny information (minimum length = 5 kb) a circos [47] plot was constructed to identify the most syntenous genomes among all (n = 18).

For identification of proteins being selected positively, dN/dS ratio was calculated in pairwise manner for the set of orthologous proteins which were further aligned using CLUSTAL W algorithm [48]. Nucleotide sequences of these alignments were then aligned codon by codon, using the PAL2NAL script [49]. The dN/dS ratio for each pair of proteins was hence calculated using Yn00 module of the PAML package [50]. Protein pairs with > 30% length difference were excluded from further analysis. To focus on the time-independent attribute of natural selection, dN/dS ratios were plotted against dS values [51].

### Identification of HGTs and MGIs (Metagenomic Islands)

Genomic islands (GIs) profile was generated using SIGI-HMM [52] at sensitivity value of 0.7 to determine transition probabilities at the gene level. For this purpose the %codon bias and % G + C difference was calculated across 2 kb window size for strain RL and its 17 phylogenetic neighbors (Additional file 1: Table S1). GIs were then annotated using BLASTX (E-value = 10^−5^) against COG [53] and KEGG database [36] for putative HGTs. Metagenomic reads were mapped over the 18 *Pseudomonas* genomes using GASSST (Global Alignment Short Sequence Search Tool) [54] at 80% sequence similarity cut-off and enabled reverse complement search. Metagenomic mapping included both whole-genome and genome-specific markers (GSMs) binning using k-mer based GSMers [55]. Metagenomic reads were hence tilled against the reference genomes (n = 18) and GSMs with a view to understand mechanism generating variability reflected by the metagenomic islands and their subsequent potential adaptations in the stressed environments. Further, metagenomic islands were predicted explicitly across strain RL genome using method previously reported by Sangwan *et al*., (2014) [16] to understand HCH stress specific enrichment as well as vestigiality of genes pertinent to strain RL.

### Statistical analysis

All the statistical computations used in this study were performed in R [56]. For comparative functional analysis, Principal Component Analysis (PCA) was performed on 131 variables (denoting the metabolic pathways) and 18 factors denoting the genomes. This was followed by hierarchical clustering on the first two dimensions of PCA using Euclidean metric and ward as method. Top 50 variables (metabolic pathways) were also plotted at 0.8% relative abundance and 0.6% variance cut-off. Species-specific abundance was analyzed with respect to HCH stress gradient via non-metric multidimensional scaling (NMDS) using the Vegan package [57] in R. NMDS analysis was also used to determine the inter-species genetic variance with respect to variable genome size, %G + C difference, %codon bias, HGT proteins and MGEs. Fisher’s exact t-test was performed using sequential Bonnferroni corrections [58] with adjusted *P*-values < 0.001, for all pairwise trait comparisons of *Pseudomonas* genomes used in this study.

### Summary of reference genomes and metagenomes

Strain RL, a HCH non-degrader, was isolated from HCH contaminated pond and was further used along with its 17 reference genotypes in comparative analysis to understand evolutionary dynamics under varied habitat specific selective pressures. For assigning phylogenomic status to strain RL, whole genome sequences from 40 representative *Pseudomonas* species were used as available in NCBI database (http://www.ncbi.nlm.nih.gov/genome/browse/representative/). Additionally, 10 strains (sub-species) were also used belonging to *P. aeruginosa* (n = 4), *P. stutzeri* (n = 4), and *P. mendocina* (n = 2) for being 10 closest phylogenetic neighbors to strain RL predicted by RAST whole genome based functional scores. Involvement of multiple strains for these species further enabled us to study intra-species functional demarcations across them. *Pseudomonas* sp. TKP [59] and *P. aeruginosa* MTB1 [60] were also included owing to their similar ecotype status as strain RL i.e. isolated from HCH contaminated soils from Japan. Phylogenomic reconstruction hence was performed using 400 conserved bacterial marker genes [39] and 50 *Pseudomonas* strains which revealed 16 *Pseudomonas* strains as phylogenetic neighbors to strain RL. These strains were used for further detailed comparative genomic analysis along with *Pseudomonas* sp. TKP which was included for inhabiting similar HCH stress (as strain RL) although an outlier from related reference genotypes as per the phylogenetic tree. Hence, these 17 *Pseudomonas* genomes were referred to as reference ecotypes/phylogenetic neighbors of strain RL. Sequence data was obtained for the *Pseudomonas* genomes from NCBI Genome database: *Pseudomonas* sp. TKP [GenBank:NC_023064.1], *P. mendocina* ymp [GenBank:NC_009439.1] *P. mendocina* DLHK [GenBank:NZ_ALKM00000000.1], *P. mendocina* NK-01 [GenBank:NC_015410.1], *P. aeruginosa* 9BR [GenBank:NZ_AFXI00000000.1], *P. aeruginosa* 19BR [GenBank:NZ_AFXJ00000000.1], *P. aeruginosa* 213BR [GenBank:NZ_AFXK00000000], *P. aeruginosa* DK2 [GenBank:NC_ 018080.1], *P. aeruginosa* MTB-1 [GenBank:NC_023019.1], *P. stutzeri* T13 [GenBank:NZ_ALJB01000000.1], *P. stutzeri* XLDN-R [GenBank:NZ_AKYE00000000.1], *P. stutzeri* CCUG 29243 [GenBank:NC_018028.1], *P. stutzeri* ATCC 14405 [GenBank:NZ_AGSL00000000.1], *P. stutzeri* A1501 [GenBank:NC_009434.1], *P. psychrotolerans* L19 [GenBank:NZ_AHBD00000000.1], *P. luteola* XLDN4-9 [GenBank:NZ_ALAT00000000.1] and *P. denitrificans* ATCC 13867 [GenBank:NC_020829.1].

HCH contaminated soil metagenomes were also used in this study for the metagenomic recruitment of *Pseudomonas* strains (n = 18) for understanding the extent of flexible genotype maintained across *in situ* cohorts under the selective pressure of HCH contamination. We used metagenomic reads [SRP 047092 under NCBI Short Read Archive] from the HCH contaminated pond sediment which is also the source of isolation for strain RL, along with metagenomic reads from a previous HCH metagenomic survey [8]; HCH dumpsite [MG-RAST ID: 4461840.3], soil that was 1 Km away [MG-RAST ID: 4461013.3] and 5 Km [MG-RAST ID: 4461011.3] away from HCH dumpsite.

## Results and discussion

### Physicochemical analysis

Physicochemical analysis of the sediment samples revealed moderately saline nature of the soil with a pH of 7.22 and electrical conductivity of 2.33 dS m^−1^ (Table 1). These characteristics were accredited to the presence of high concentration of ions, particularly cations including phosphorous (61.60 kg ha^−1^), potassium (1254.50 kg ha^−1^) [61], and nitrogen (249.50 kg ha^−1^) (Table 1) [62]. Comparison with previously surveyed similar HCH contaminated soils i.e. HCH dumpsite (918.00 kg ha^−1^), 1 Km (40.50 kg ha^−1^) away and 5 Km (84.30 kg ha^−1^) away soil samples from the dumpsite [8], HCH contaminated pond soil (1254.50 kg ha^−1^) was found to be the most enriched in available potassium implying at it’s *in situ* bioremediation potential [61] (Table 1). Relatively low HCH concentration (i.e. 45 mg HCH g^−1^) estimated in pond samples in comparison to HCH dumpsite (i.e. 450 mg HCH g^−1^) [8], can be attributed to the low solubility of HCH isomers in water i.e. 2 mg L^−1^, 0.2 mg L^−1^, 7.3 mg L^−1^, and 31.4 mg L^−1^ for α-, β-, γ-, and δ-HCH respectively [63].

**Table 1:** **Physicochemical analysis of HCH contaminated pond sediment and soil samples**

**Characteristics**	**Pond_1**	**Pond_2**	**Pond_3**	**Pond_4**	**Dumpsite**	**1 Km**	**5 Km**
**pH**	7.24	7.20	7.19	7.23	7.21	7.81	7.93
**EC (dS m** ^**−1**^ **)**	2.35	2.3	2.25	2.43	8.50	0.19	0.43
**Organic Carbon (%)**	1.54	1.48	1.62	1.53	30.74	0.45	0.67
**Available P (kg ha** ^**−1**^ **)**	61.1	62	61.5	61.8	60.3	93	318
**Available K (kg ha** ^**−1**^ **)**	1,254	1,260	1,255	1,249	918	40.5	84.3
**Available N (kg ha** ^**−1**^ **)**	251	250	248	249	335	397	460
**ΣHCH (mg g** ^**−1**^ **)**	45	43	41	51	450	0.7	0.03
**Remarks**	Saline	Saline	Saline Soil	Saline	Saline	Normal	Normal
	Soil	Soil		Soil	Soil		

**ΣHCH:** sum of α and β HCH isomers.

### General features of *Pseudomonas* genomes

**Figure 1:**
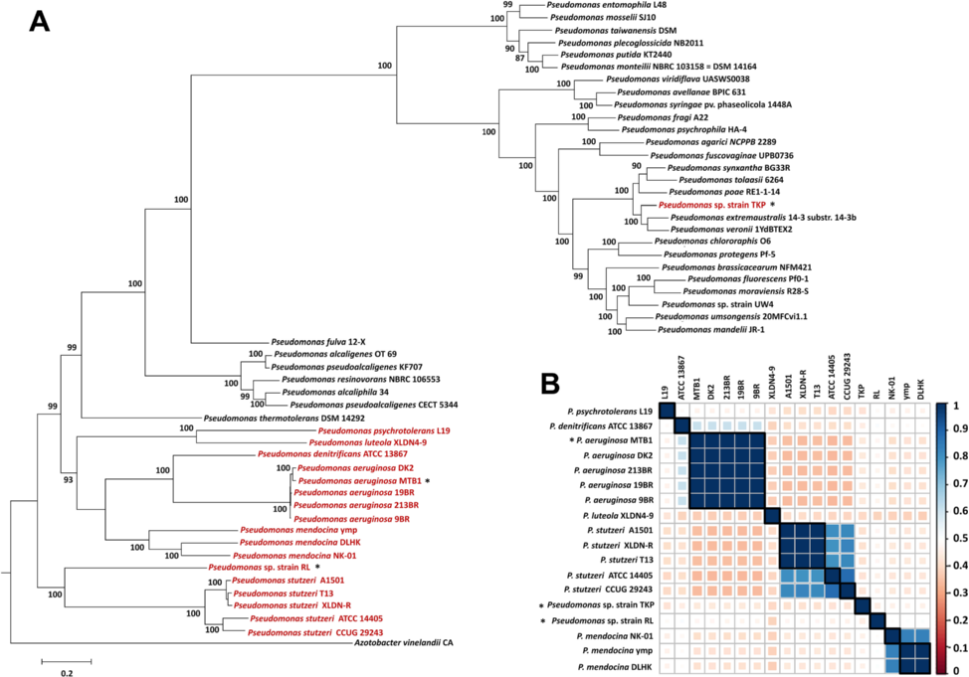
Phylogenomic analysis of 18 *Pseudomonas* genotypes. **(A)** Conserved genes (400) based phylogenetic tree of strain RL and representative *Pseudomonas* genotypes (n = 51) constructed at 1000 bootstrap with *Azotobacter vinelandii* CA as an out-group. Strains labeled in red color are the ones chosen for the comparative study and those labeled with asterisks (*) are the ones inhabiting HCH contaminated soils. Branch lengths are drawn to scale, with scale bar indicating the number of amino acid substitutions. Numbers on branches are the bootstrap values of the clusters on the right. (**B)** Correlation plot for *Pseudomonas* sp. strain RL and its 17 reference genotypes on the basis of pairwise ANI values. High (blue) and low (pink) correlation and predicted correlation coefficients (0 to 1.0) are shown with the color scale on the basis of respective correlation values.

Raw sequence data (Illumina HiSeq 2000 = 1.3 Gb (pair-end) and 454 GS FLX = 53.5 Mb (single-end)) generated for strain RL was assembled into contigs (n = 228, > 500 bp) with N50 of 22,320 bp and maximum contig length of 1,00,097 bp using Velvet_1.2.03 [29]. The automated annotation of *Pseudomonas* sp. RL (3.8 Mb) using RAST [40] revealed 3,499 protein coding sequences, 466 subsystem features and 65% of G + C content. To establish correlation, 17 reference genotypes of strain RL as determined by phylogenomic reconstruction (Figure 1A) were also annotated simultaneously. Strain RL was found to be the smallest (3,813,159 bp) with strain TKP to be the largest of all (7,012,672 bp) (Table 2), which was also reflected by the number of protein coding sequences predicted for strain RL (n = 3,499) and *Pseudomonas* sp. TKP (n = 6,314) (Table 2).

**Table 2:** **General genome features of** ***Pseudomonas*** **sp. RL and its 17 reference genotypes**

**Genome**	**Genome size (in bp)**	**%G + C**	**rRNA**			**tRNA**	**Presence of integron associated integrase**	**No. of annotated proteins**	**HGT proteins**	**Orthologous proteins**	**Habitat and accession numbers for genome sequences**
			**5S**	**16S**	**23S**						
***Pseudomonas*** **sp. RL**	3,813,159	65	1	2	3	39	Present	3,499	294	*	HCH dumpsite, India (JBOY00000000)
***P. aeruginosa*** **19BR**	6,742,964	66	4	4	4	74	Present (99%, E = 0.0)	6,163	592	817	MDR strain (clinical) (NZ_AFXJ00000000.1)
***P. aeruginosa*** **213BR**	6,719,211	66	4	4	4	74	Present (99%, E = 0.0)	6,148	129	815	MDR strain (clinical) (NZ_AFXK00000000)
***P. aeruginosa*** **9BR**	6,849,861	66	4	4	4	74	Present (99%, E = 0.0)	6,296	603	817	MDR strain (clinical) (NZ_AFXI00000000.1)
***P. aeruginosa*** **DK2**	6,402,658	66	4	4	4	76	Absent	5,883	182	815	Cystic fibrosis patient (clinical) (NC_ 018080.1)
***P. aeruginosa*** **MTB-1**	6,580,038	66	4	4	4	63	Absent	6,095	223	810	HCH contaminated soil, Japan (NC_023019.1)
***P. stutzeri*** **ATCC 14405**	4,525,589	61	1	1	1	53	Absent	4,124	363	851	Polluted marine isolate, denitrifying bacterium (NZ_AGSL00000000.1)
***P. stutzeri*** **CCUG 29243**	4,709,064	63	4	4	4	64	Absent	4,300	284	787	Polluted marine isolate, naphthalene degrading (NC_018028.1)
***P. stutzeri*** **XLDN-R**	4,695,416	64	2	2	2	64	Absent	4,336	401	836	Soil, carbazole degrading (NZ_AKYE00000000.1)
***P. stutzeri*** **T13**	4,648,939	64	1	1	1	52	Present (99%, E = 0.0)	4,361	397	827	Sludge (NZ_ALJB01000000.1)
***P. stutzeri*** **A1501**	4,567,418	64	4	4	4	66	Absent	4,217	476	827	Rhizoshphere (NC_009434.1)
***P. mendocina*** **ymp**	5,072,807	65	4	4	4	71	Absent	4,594	424	778	Yucca mountain sediment, high level nuclear respository (NC_009439.1)
***Pseudomonas mendocina DLHK***	5,072,093	64.7	1	1	1	53	Absent	4,621	467	598	Bioreactor, denitrifying bacteria (NZ_ALKM00000000.1)
***Pseudomonas mendocina NK-01***	5,434,353	62.5	4	4	4	65	Absent	4,958	345	641	Farmland soil, China (NC_015410.1)
***Pseudomonas psychrotolerans L19***	5,100,236	65.7	1	1	1	57	Absent	4,759	387	781	50 percent copper alloy coin (NZ_AHBD00000000.1)
***Pseudomonas luteola XLDN4-9***	4,626,799	54.2	Not found	1	1	46	Absent	4,299	266	643	Soil (NZ_ALAT00000000.1)
***Pseudomonas denitrificans ATCC 13867***	5,696,307	65.2	6	5	5	63	Absent	5,056	103	792	Not found (NC_020829.1)
***Pseudomonas*** **sp. TKP**	7,012,672	61	6	5	5	68	Absent	6,314	314	814	HCH contaminated soil, Japan (NC_023064.1)

Values in cells with *were not determined as orthologous protein determination was done with respect to *Pseudomonas* sp. RL.

**Figure 2:**
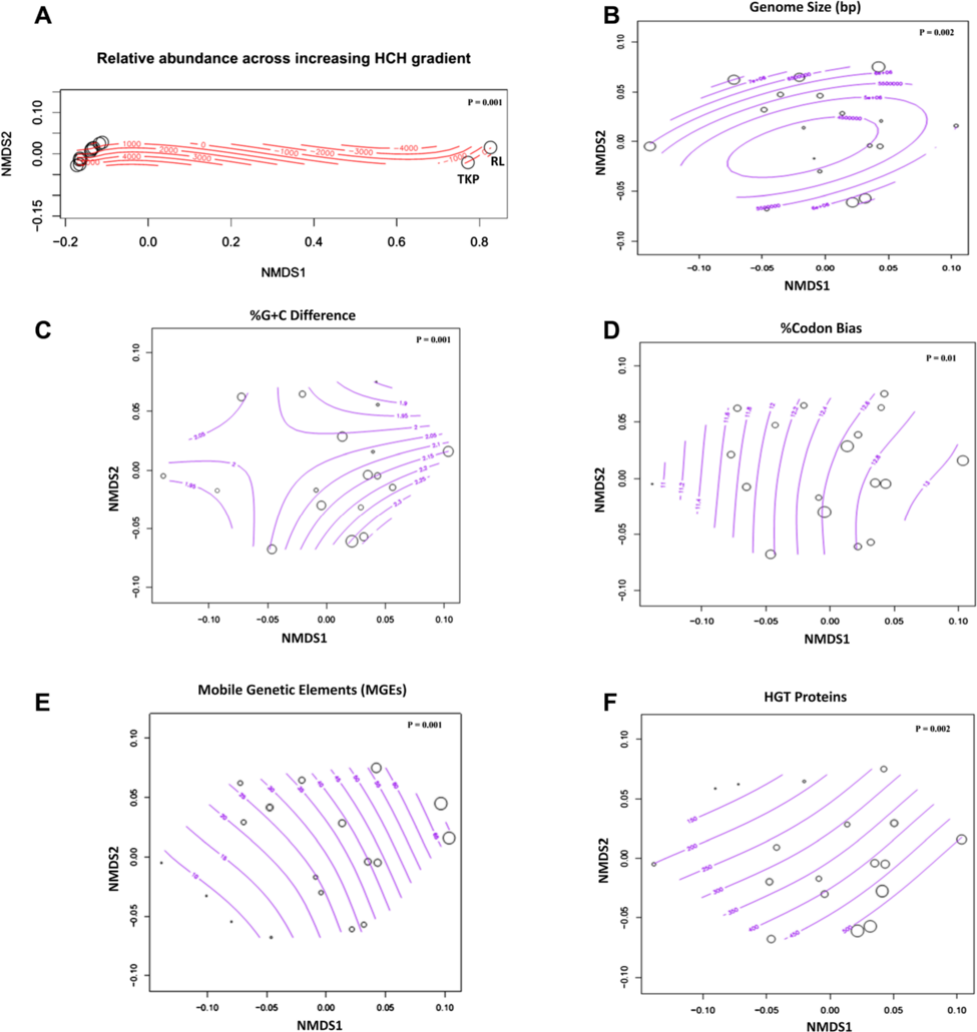
Non-metric dimensional scaling (NMDS) ordination analysis. **(A)** Relative abundance of *Pseudomonas* species (n = 18) enriched across increasing HCH gradient. **(B)-(F)** Species abundance with respect to genome characteristics; genome size, %G + C difference, %codon usage bias, HGT proteins, and mobile genetic elements, respectively. Bubble size and contour lines represents abundance.

NMDS analysis of %G + C difference and %codon bias patterns revealed significantly (*P*-value > 0.002) similar inter-species genetic variance (Figure 2). The %G + C difference and %codon bias values for strain RL (%G + C difference = 0.018, %codon bias = 0.114), strain TKP (%G + C difference = 0.016, %codon bias = 0.118) and strain MTB1 (%G + C difference = 0.018, %codon bias = 0.093) clustered in a close range compared to the other reference genotypes (Additional file 1: Table S1). Clustering patterns indicated at genetic coherence in terms of adaptation towards similar habitats (HCH stressed soils) as compared to other genotypes inhabiting marine or soil/sediments. It has also been established for pseudomonads to demonstrate a relatively high codon usage bias because of the necessity to acclimatize diverse environments [64].

### Phylogenomic analysis

We used a set of 400 conserved bacterial marker genes to determine the phylogenetic status of strain RL. A phylogenetic tree was constructed using maximum likelihood methodology revealing prominently three major groups neighboring strain RL, including *P. stutzeri*, *P. aeruginosa,* and *P. mendocina* (Figure 1A). Strain RL fell within the cluster including *P. stutzeri* group though delineated at species level (ANI ≤ 80.03%). Above strain RL, a larger cluster was observed containing two sub-groups of *P. aeruginosa* and *P. mendocina* along with presence of individual representative strains for *P. denitrificans* (ATCC 13867), *P. luteola* (XLDN4-9), and *P. psychrotolerans* (L19) (Figure 1A). Comparing the intra-species phylogenetic topology of two major groups of *P. aeruginosa* and *P. stutzeri*, *P. aeruginosa* genomes were found to form a tight monophyletic clade in contrast to largely divergent *P. stutzeri* group (Figure 1A). These results were in good agreement with ANI analysis, where pairwise ANI values for *P. aeruginosa* members (n = 5; 19BR, 213BR, 9BR, DK2, MTB1) were greater than 99% suggesting these strains to be sub-species (Figure 1B) [65]. However, two sub-groups were identified for *P. stutzeri* clade, first with *P. stutzeri* CCUG 29243 and ATCC 14405, and second containing *P. stutzeri* A1501, T13 and XLDN-R (Figure 1A). This distinct deviation could be accredited to the ecotype status resulting from the niche specific adaptations, as *P. stutzeri* ATCC 14405 and CCUG 29243 both are marine isolates [66,67] however, A1501, T13, and XLDN-R, are well-known soil/sludge dwellers [68,69]. ANI values for *P. stutzeri* group (≤88.2%) were also not in accordance with species demarcation value of 95-96% [65] indicating towards intra species level divergence (Figure 1B). *P. mendocina* ymp along with other *P. mendocina* strains was placed outside both *P. aeruginosa* (ANI ≤ 79.98%) and *P. stutzeri* (ANI ≤ 79.97%) groups (Figure 1B). *P. denitrificans* ATCC 13867 (ANI ≤ 82.08%), *P. luteola* XLDN4-9 (ANI ≤ 76.42), and *P. psychrotolerans* L19 (ANI ≤ 79.01) were revealed to be organized in the same major clade alongside *P. aeruginosa* and *P. mendocina* but as separate nodes demonstrating species delineation supported by ANI values as well (Figure 1A, B).

### Structure and characterization of integron

**Figure 3:**
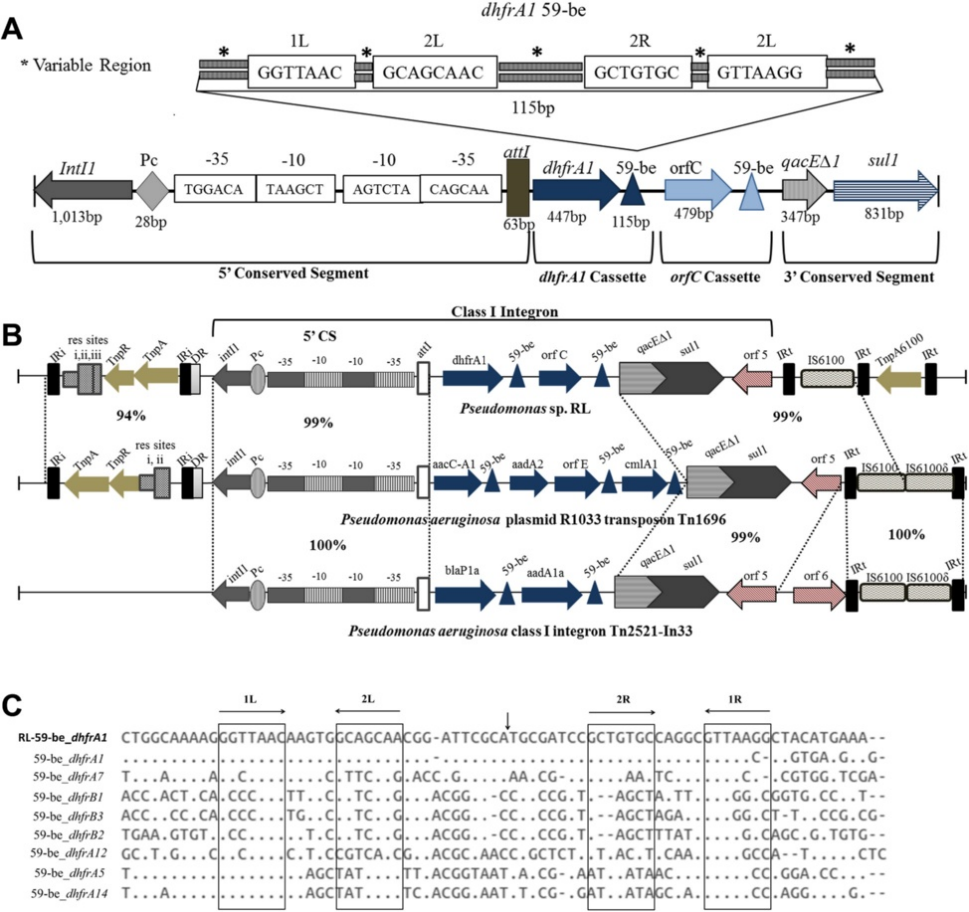
Structure and characterization of Integron. **(A)** Schematic representation of class-I integron with the gene cassette structure as determined in RL. Horizontal arrows show the gene orientation. **(B)** Schematic representation of comparison of class-I integron element between RL, *P. aeruginosa* plasmid R1033 transposon Tn1696 [GenBank:U12338.3], and *P. aeruginosa* class I integron Tn2521-In33 [GenBank:AF313471.1]. % Identity between the integron elements is based on BLASTN results at E-value cut-off = 1e-5. Horizontal arrows show the gene orientation. IRi and IRt stand for invert repeats. **(C)** Alignment results of 59-be element associated with *dhfra1* gene in strain RL (topmost) with already determined classes of *dhfr* gene [72]. Vertical arrow indicates the intermediate variable sequence not shown in the alignment.

RAST server 4.0 annotation revealed presence of *intI* gene on one of the contigs of strain RL, in turn indicating at probable presence of an integron. Further, BLASTN analysis of this contig against INTEGRALL 1.2 database [38] predicted a complete class-I integron containing gene cassette flanked by 5’ Conserved Site (CS) and 3′-CS [70]. We identified (Figure 3A) 5′-CS containing DNA integrase gene (*IntI1*) (1,013 bp) belonging to tyrosine-recombinase family, a promoter site (Pc) (28 bp) for cassette associated genes, followed by promoter for the integrase (Pi) and *attI* site (63 bp) which is the site of integrase driven recombination [70] (Figure 3A). 3′-CS was found to contain 2 genes: *sul1* (831 bp) conferring sulfonamide resistance and *qac1* (347 bp) encoding for protein that confers resistance against quaternary ammonium compounds (Figure 3A). This observation was in accordance with the extent of 3′-CS illustrated by Stokes *et al*. in 1989 [70]. Presence of *sul1*gene (Figure 3A), in the 3′-CS site speculates a site-specific insertion event in past, which led to its integration into the ancestral integron element [70].

The gene cassette inserted between 5′-CS and 3′-CS was found to be encoding dihydrofolate reductase (*dhfra1*) (447 bp) gene which confers trimethoprim resistance (Figure 3A). A 115 bp long 59-be (base element) was found downstream of *dhfrA1* gene containing 2 short stretches of complete conservation designated as L (left) and R (right) [71] (Figure 3C). Each of these sites was found to be made up of a pair of inversely oriented *intI* binding domains known as 1 L, 2 L, 1R, and 2R separated by spacers (Figure 3C). We determined a 7 bp long 1 L (GGTTAAC) and 8 bp long 2 L site (GCAGCAAC), separated by a 5 bp spacer. Similarly, a 7 bp long 1R (GTTAAGG) and 2R site (GCTGTGC) were also determined with a 5 bp long spacer. 2 L and 2R sites were found to be separated by a 39 bp sequence consistent with *dhfra1* associated 59-be. Alignment of 59-be elements associated with different classes of *dhfr* gene i.e. *dhfrA1*, *dhfrA5, dhfrA7, dhfrA12, dhfrA14, dhfrB1, dhfrB2,* and *dhfrB3* revealed that 1 L, 2 L, 2R sites were identical to those associated with *dhfrA1* gene but 1R site showed a minor change in this case. 1R site in strain RL was identified to be i.e. GTTAA’GG’ (7 bp) instead of GTTAA’CC’ (Figure 3C). The alignment also revealed that the last 3 residues (‘AAC’) in 1 L site and first 3 residues (‘GTT’) in 1R site are completely conserved (Figure 3C). The *intI* facilitated recombination is documented to be restricted between the G and TT region in 1R of the 59-be [72], which was found to be completely intact in RL’s integron unit. Integrons though have been found in different genera but very few have been reported in *Pseudomonas* spp. [22] and rarer is the *dhfrA1* gene association with class I integron.

Finally, detailed comparison of integron unit of strain RL with its phylogenetic neighbors revealed presence of an intact integron in *P. aeruginosa* 19BR and 213BR though no association with transposon (Additional file 2: Figure S1) was observed. However, in case of *P. stutzeri* T13, a mobile integron linked with transposon was determined that has yet to capture gene cassette (Additional file 2: Figure S1).

### Environment specific activation of class-I integron revealed through metagenomics

Evidence for the mobility of integrons is their association with transposons, insertion sequences (ISs) or conjugative plasmids [73]. Elaborate study of the reconstructed integron elucidated presence of conserved 5′-CS and 3′-CS sites and a gene captured in between, which indicated at the separation event of these elements. In this study, we confirmed the presence of class-I integron (3,552 bp) in RL on a Tnp21-like transposon (BLASTN; 95%, E-value = 0.0) (Figure 3B). The transposon (Tn) module was found to be present upstream (100 bp) in the beginning of the integron. The module consisted of ORFs encoding for resolvase specific sites i.e. res I (9 bp), res Ii (27 bp), res III (32 bp), and TnpR (615 bp) and TnpA (2,967 bp) and was found to be flanked by IR (invert repeat sequences) on both sides (Figure 3B). This was then followed by a complete integron adjoined by a TnpA6100 element at its 3′ end, indicating its potential to perform active gene transfer events.

**Figure 4:**
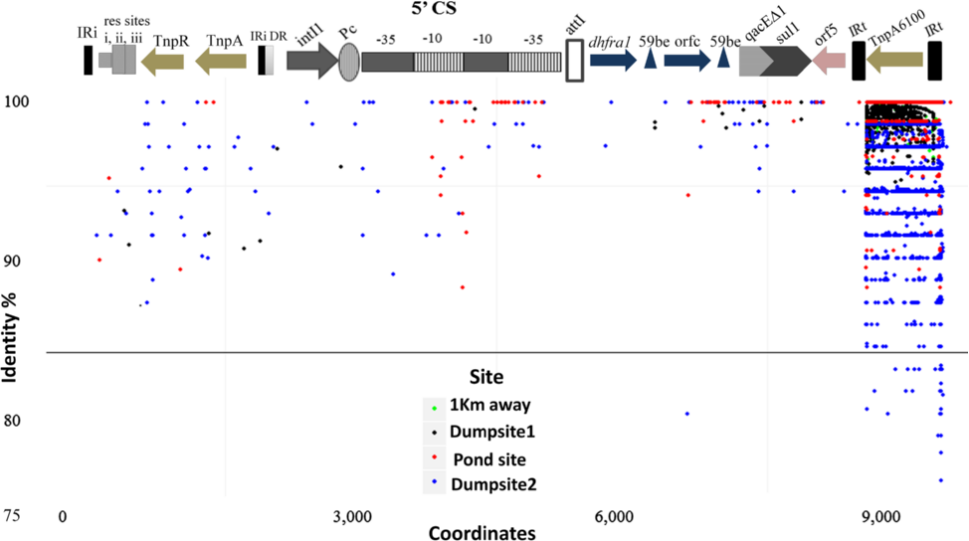
Metagenomic recruitment of Class-I integron. Recruitment plot showing mapping of metagenomic reads from HCH contaminated pond and previous metagenomic survey data [8] from HCH dumpsite (Pyro), HCH dumpsite (Illumina) and 1 Km away samples, on class-I integron of strain RL. A dot represents each read aligned onto the integron, with color coding for respective metagenome sites. *x* and *y* axis represent the sequence co-ordinates and sequence identity, respectively.

Metagenomic recruitment (global alignment identity cut-off = 80%) revealed concentrated mapping for IS*6100* associated transposase-TnpA6100 gene, specifically in HCH dumpsite metagenome (illumina) data [8], highlighting it as an environment specific character (Figure 4). Since, TnpA6100 is well established for carrying out inter and intra genome DNA transfers [23], our results here implied at species-specific mobility of class-I integron in strain RL enriched across the HCH contamination. TnpA6100 was also subjected to BLASTN search against NCBI-nt database, revealing best representatives from *Klebsiella pneumonia* (Identity% = 100, E-value = 10^−10^) and *E. coli* (Identity% = 100, E-value = 10^−9^) validating the foreign origin of TnpA6100 in RL. The *in situ* enrichment of this transposase can be attributed to a stress response of *Pseudomonas* cohorts under HCH pressure, although the exact (species-specific) mechanism is yet unknown. However, a targeted (enriched) and deeply sequenced community genomics survey in combination with increased number of reference genome sequences can resolve the actual *in situ* mechanism of integron mediated gene transfers at a higher resolution i.e. in terms of gene frequencies and sequence variations.

### Comparative metabolic capabilities

**Figure 5:**
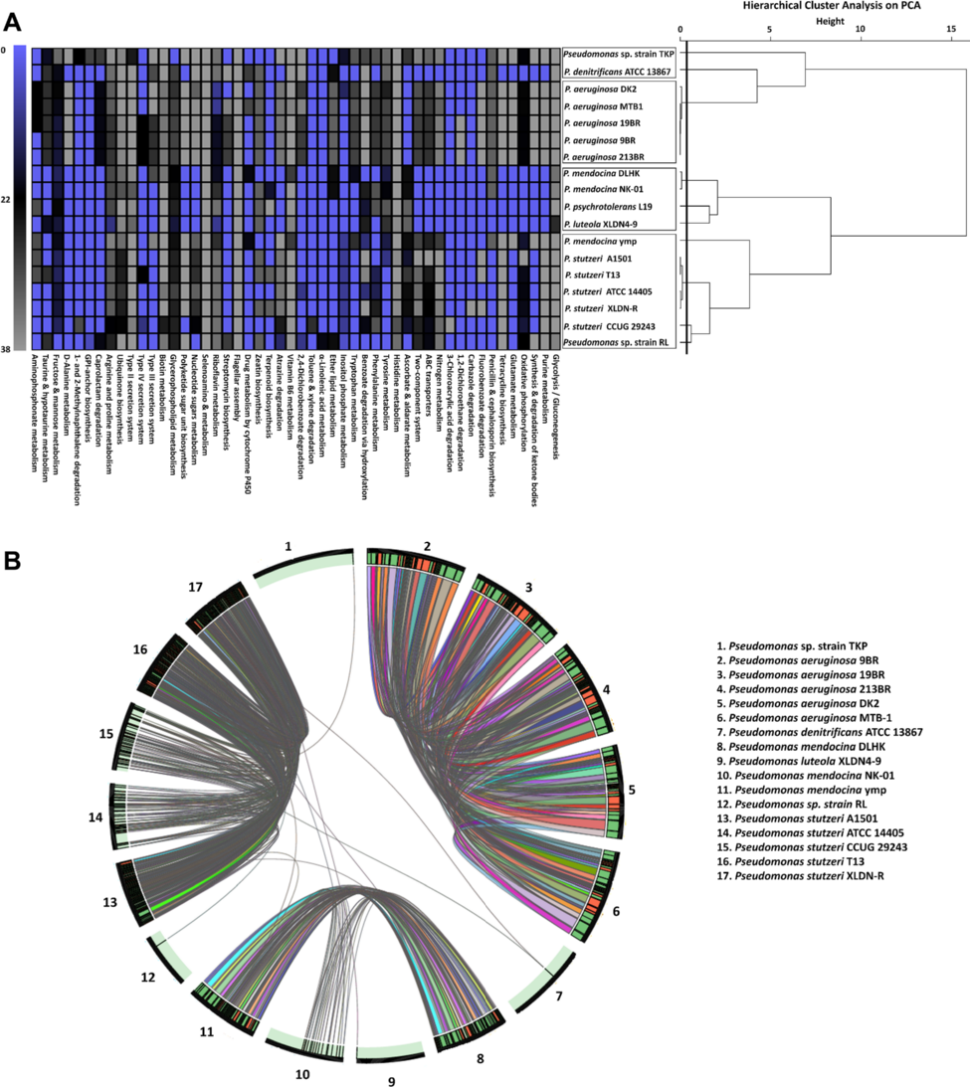
Comparative functional potential across 18 *Pseudomonas* strains. **(A)** Hierarchical cluster tree based on the PCA analysis of 131 metabolic pathways reconstructed in strain RL and its 17 reference genomes. Heatmap showing family level reconstruction of top 50 metabolic pathways among 18 *Pseudomonas* genomes in this study (percentage cut-off = 0.8%, variance cut-off = 0.6%). **(B)** Whole genome synteny plot of *Pseudomonas* sp. RL and its 17 phylogenetic neighbors (Table 2). A window size of 5 kb was used. Numbers 1–17 are labeled according to the legend provided on the right corner. Colored blocks (red/green) for each genome’s base represents their orientation; green = positive, red = negative. Connecting arcs are drawn in grey color.

HCPC (Hierarchical Clustering on Principal Components) analysis on reconstructed metabolic pathways revealed two major clusters (Figure 5A). The first cluster consisted of strain RL, *P. psychrotolerans* L19, *P. luteola* XLDN4-9 and multiple strains from *P. stutzeri* and *P. mendocina.* However, the second cluster had an intact sub-cluster of *P. aeruginosa* strains wit*h strain* TKP and *P. denitrificans* ATCC 13867 falling out (Figure 5A) of the sub-cluster. These results were partially in congruence with phylogenetic analysis i.e. *P. aeruginosa* strains making a tight cluster unlike *stutzeri* strains which showed distinct branching patterns within the cluster (Figure 1A and B). These observations highlighted the functional variances among *P. stutzeri* strains (Figure 5A) which we have attempted to analyze at higher resolution with respect to the ecotype deviation. Agreement between metabolic and phylogenetic analysis is significant here as the concept of deriving organismal phylogeny using universal proteins can be biased as it represents only the well conserved proteins hence offering skew in case of genomes with substantial amount of HGTs [74] (Figure 5A). Therefore, we have substantiated our phylogenetic analysis with functional insights which included both core and flexible genome (HGT candidates) (Figures 1A, B and 5A).

KEGG based metabolic pathway reconstruction was used to compare the functional repertoire of *Pseudomonas* genomes (n = 18, Table 2). The central metabolic pathways such as glycolysis and pentose phosphate pathway in strain RL were in sync with those reported in its phylogenetic neighbors (n = 17). However, in contrast to all of the *Pseudomonas* genomes used in this study strain RL revealed unique presence of α-linoleic acid degradation pathway. Although, multiple *Pseudomonas* species (e.g. *fluorescens, putida, alcaligenes*) have been reported to degrade the fatty acid derivatives, its implicit presence in RL indicates at its differential capacity to use α-linoleic acid as carbon source [75]. Similarly, when compared to the other genomes (n = 17), *P. stutzeri* XLDN-R was found to exclusively possess the carbazole degradation pathway which was further supported by the fact that this particular strain was isolated from soil for its differential ability to utilize carbazole as sole source of both carbon and nitrogen [69]. 3-chloroacrylic acid degradation pathway was found uniquely in *P. stutzeri* CCUG 29243 indicating at its capability of using 3-chloroacrylic acid as carbon source [76]. In addition, toluene & xylene degradation potential was found to be an exclusive metabolic feature of *P. stutzeri* CCUG 29243 genome, which further highlighted towards its bioremediation potential against toluene contamination [77].

Pairwise functional comparisons (pathways) performed across *P. aeruginosa* (n = 5) and *P. stutzeri* (n = 5) strains revealed the genetic predominance of 2,4-dichlorobenzoate degradation and ether-lipid metabolism in *P. aeruginosa* strains. Presence of 2,4-dichlorobenzoate degradation pathway supported the existence of an efficient enzyme system for chlorobenzoate degradation already established for *P. aeruginosa* strains [78]. In contrast to *P. stutzeri* strains the *P. aeruginosa* strains were also found to possess the streptomycin degradation pathway highlighting its genetic potential of being a persistent opportunistic pathogen i.e. extra-ordinary capability to inactivate multiple aminoglyocosides including streptomycin [79]. As reported in a previous study, caprolactam degradation [80] and D-alanine catabolism pathways are the distinct features of *P. aeruginosa* strains. Interestingly, *P. aeruginosa* strains can preferentially catabolize D-alanine over other carbon sources to promote the production of extra-cellular virulence factors [81]. This preferential acquisition of multiple functional traits (pathways) in *P. aeruginosa* strains in contrast to other pseudomonads reveals the persistent evolutionary processes undertaken to evolve as a metropolitan pathogen [81].

KEGG metabolic pathway based analysis further revealed several lifestyle specific preferences (secretion systems) across pseudomonad genomes (n = 18). As for an example, *P. aeruginosa* strains (known human pathogens), were revealed to possess the complete genetic repertoire for Type III (T3SS) (13/15 KOs) and Type IV (T4SS) secretion systems (10/12 KOs). Presence of type III and IV secretion systems clearly indicates at the pivotal metabolic role of secretion systems in *P. aeruginosa* strains (clinical superbugs) in both secretion and injection of virulence factors into the host environments [82]. However, *P. stutzeri* strains (nonpathogenic; KOs = 0) were completely devoid of genes related to Type III and IV secretion system. Interestingly, several intra-species (niche-specific) metabolic adaptations were also revealed between the *P. stutzeri* ecotypes i.e. marine and soil/sediment isolates. Marine inhabitants i.e. ATCC 14405, CCUG 29243 in contrast to other *stutzeri* strains (soil dwellers) were found to lack genes involved in fluorobenzoate degradation [83] and zeatin biosynthesis pathways, which already have been established as the characteristic features of soil dwelling pseudomonads e.g. zeatin helps in inter-cell communication with plants [84].

Detailed functional analysis was performed explicitly between HCH tolerant pseudomonads i.e. strain RL, TKP and *P. aeruginosa* MTB1. Association of *lin* genes with HCH degradation has already been studied in detail in sphingomonads [17-20,85-89]. In contrast to sphingomonads, both the upper (*linA, linB, linC*) and lower pathway genes (*linDER*, *linKLMN*, *linGHIJ*, and *linF*) required for γ-HCH degradation [17] were absent among these HCH inhabitants (pseudomonads). To rule out the altercations conferred by low sequence coverage and assembly we established the complete absence of *lin* genes by comparing both contigs (validated by paired end criterion) and ORFs (contigs = BLASTX and ORFs = BLASTP) against the reference *lin* gene database [8]. In addition, the quality filtered raw sequence reads were mapped using bwa-0.5.9 [90] and BLASTN against the CDS sequences of *lin* gene database with a minimum coverage cut-off = 100X [16]. Higher *in situ* abundance and confirmed absence of HCH degradation pathway genes in these *Pseudomonas* genomes pointed towards the presence of a yet unknown metabolic way to cope up with such high HCH concentrations (45 mg HCH g^−1^ of soil). Therefore, we further focused our effort to analyze the orthologous segments between these three genomes i.e. RL, TKP, and MTB1. The detailed annotation of 1.3 Mb of orthologous segment from these HCH-tolerating *Pseudomonas* genotypes revealed (Additional file 3: Table S2) the presence of bacterial chemotaxis, two-component system, flagellar assembly proteins, and Type II secretion system along with the central metabolic pathways like TCA, glycolysis, ubiquinone synthesis, and amino acid biosynthesis. Although, we didn’t observe any novel mechanism of HCH tolerance across orthologous segments, the orthologous delineation of chemosensory two-component system (*pilG*, *pilH*, *cheB*, *cheY*, *fleR*, *phoB*, and *gacA*) across RL, TKP and MTB1 highlighted towards the importance of this system in genetically adapting against HCH stress (Additional file 3: Table S2).

### Identification of orthologues and proteins under positive selection

We used a two-step based approach for the identification of orthologous proteins across all 18 *Pseudomonas* genomes. First step i.e. whole genome based multiple alignments using Murasaki [44] and OSfinder v1.4 [45], revealed protein families including but not limited to; two-component system, DNA replication associated proteins, ribosomal subunit forming proteins, recombinase, bacterioferritins, flagella associated proteins, electron-transferring dehydrogenases, cell division proteins, transcription repair proteins as core features across pseudomonad genomes (Additional file 4: Table S5).

In second step i.e. pairwise protein profile comparison, the RSD analysis [46] was performed between RL and its phylogenetic neighbors (n = 17). RSD analysis predicted maximum of 851 orthologous proteins for *P. stutzeri* ATCC 14405 and minimum of 598 proteins for *P. mendocina* DLHK, with maximum likelihood distance estimate > 2 (Table 2). A common set of pairwise orthologous proteins were further implemented for dN/dS analysis to identify the positively selected functions. In order to study the inter-species evolutionary dynamics, orthologs were also identified between *P. stutzeri* and *P. aeruginosa* genomes. Both the groups (10 strains (5 each of *aeruginosa* and *stutzeri*)) shared a total of 681 orthologs (442 unique KO entries) which included functional pathways like pilus assembly, septum site determination, DNA replication, ribosomal assembly, multiple sugar transport, recombination, flagellar assembly, transcriptional regulation, membrane proteins and chemotaxis (Additional file 5: Table S6).

In addition, a genome-wide synteny analysis (window size cut-off = 5 kb) was also performed for 18 genomes, which highlighted (Figure 5B) the higher levels of genome wide synteny between *P. aeruginosa* genomes (19BR, 213BR, 9BR, DK2, MTB1) i.e. larger (>5 kb) conserved blocks, in contrast to *P. stutzeri* genomes. However, strain RL and rest of the genomes showed very rare synteny events especially *P. psychrotolerans* L19 exhibiting virtually no syntenous regions at 5 kb window size, which was hence removed from the plot (Figure 5B).

**Figure 6:**
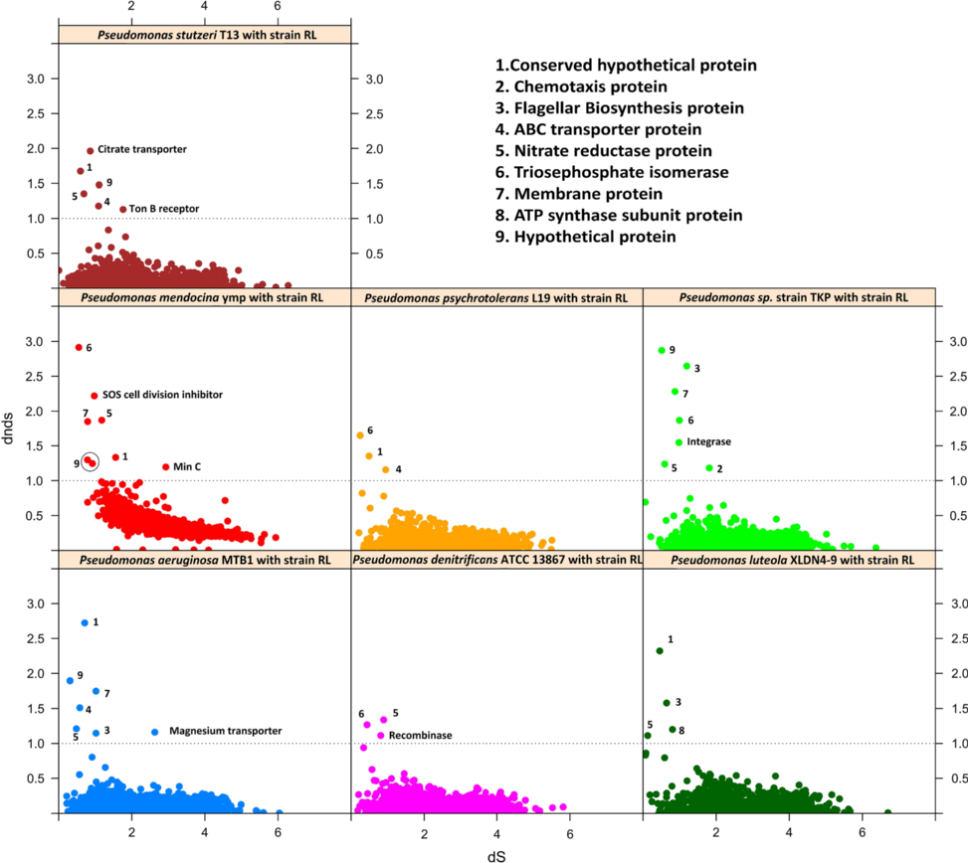
Detection of positively selected proteins. Scatter plots for dN/dS values for orthologous proteins in independent pairs of strain RL versus *P. stutzeri* T13, *Pseudomonas* sp. strain TKP, *P. aeruginosa* MTB1, *P. mendocina* ymp, *P. psychrotolerans* L19, *P. luteola* XLDN4-9, and *P. denitrificans* ATCC 13867. Black dotted line at dN/dS value of 1 represents the baseline criterion for positive natural selection. Broad functional categories are indexed from 1–9 determined in majority. Unique functions deciphered in each pair are labeled directly near the dot.

Finally, pairwise dN/dS analysis was performed for the orthologues (CDS pairs with < 30% length variation) between RL and seven representative references genomes; *P. stutzeri* T13, *Pseudomonas* sp. TKP, *P. aeruginosa* MTB1, *P. mendocina* ymp, *P. luteola* XLDN4-9*, P. denitrificans* ATCC 13867, and *P. psychrotolerans* L19. Use of these reference genomes was focused to select a representative genome from every *Pseudomonas* species involved in this study. Pairwise dN/dS analysis revealed (Figure 6) that proteins associated with chemotaxis, flagellar biosynthesis, ABC transporters, nitrate reductase proteins, membrane proteins, and conserved hypothetical proteins as positively selected across all pairwise comparisons with an average dN/dS values of 2.47, 1.36, 1.53, 1.48, 1.82, and 3.60, respectively. In addition to the above mentioned proteins, pairwise dN/dS analysis also revealed unique positive selection for citrate transporter (dN/dS = 1.19) and Ton-B receptor protein (dN/dS = 1.15) in *P*. stutzeri T13 and SOS cell division inhibitor (dN/dS = 2.24) along with MinC protein (dN/dS = 1.09) for *P. mendocin*a ymp. Similarly, magnesium transporter (dN/dS = 1.11), recombinase (dN/dS = 1.04), and integrase (dN/dS = 1.53) were found to be under positive selection exclusively in *P. aeruginosa* MTB1, *P. denitrificans* ATCC 13867, and strain TKP, respectively (Figure 6).

Interestingly, majority of these positively selected protein pairs (dN/dS > 1) have already been known to play important metabolic functions across pseudomonads genotypes. For instance, *Pseudomonas* strains are well known to utilize nitrate for both growth and to generate proton motive force (for mobility) using nitrate reductase which is initiated by uptake of nitrate via ABC transporters [91]. Similarly, flagellar biosynthesis and chemotaxis response regulator proteins work together as a unit aiding pseudomonads in both colonization and virulence [92]. Citrate transporter was found to be uniquely favored in *P. stutzeri* T13, which is established to help *Pseudomonas* strains (e.g. *P. aeruginosa*) in iron uptake via its iron-chelating activity as a siderophore [93]. Ton-B transporters were also revealed to be under positive selection in strain T13 which again helps in iron uptake, indicating at the persistent evolution of iron uptake system [94]. Integrase (recombinase) was also found to be under positive selection in strain TKP (HCH tolerant) which once again supports the role of integrase in enhancing genome plasticity under stressed conditions such as HCH contamination [23]. This also stands true for the positive selection of SOS regulon in *P. mendocina* ymp which was isolated from a nuclear waste repository characterized by enormous heavy metal stress [95]. Similarly, MinC was also found to be under positive selection which along with ATPase MinD is implied to be involved in regulating the cell growth of *Pseudomonas putida* under phenol induced stress [96]. Magnesium transporter was discovered to be under positive selection in strain MTB1, which is known to inhibit the T3SS, hence attenuating the virulence capability and also in transporting magnesium [97]. Overall, our pairwise dN/dS results indicate at the continuous genetic evolution in order to improve the genetic fitness of this taxon across stressed environments.

### Genome wide identification and annotation of HGT events

Potential HGT candidate genes were identified across 18 *Pseudomonas* genomes using %codon bias and %G + C difference analysis, implemented in SIGI-HMM program [52]. A maximum of 603 and minimum of 103 HGT proteins were predicted for *P. aeruginosa* 9BR and *P. denitrificans* ATCC 13867, respectively. The potential HGT candidates were then assigned KO numbers using KAAS, majority (>65%) of which were annotated to be the uncharacterized proteins without any assigned KO entries. Pathways were further mapped to individual proteins (with assigned KOs) using MinPath [34] (Additional file 3: Table S3). We found that RL and *P. stutzeri* A1501 revealed presence of an uncharacterized protein yhgF (K06959) potentially imported in their genomes through lateral gene transfer. *P. mendocina* ymp and *P. stutzeri* CCUG 29243 revealed methionine metabolism and one carbon pool by folate pathway to have been acquired via HGT (Additional file 3: Table S3). *P. stutzeri* ATCC 14405, *P. stutzeri* XLDN-R, and *P. stutzeri* T13 exhibited lateral acquisition of genes involved in nitrogen metabolism and TCS pathway (Additional file 3: Table S3). Interestingly, erratic incidence of nitrogen fixing genes in *Pseudomonas* spp. has always raised debate in the past and HGT has been postulated as one of the main reasons behind their acquisition [98] (Additional file 3: Table S3). Our results suggested that the evolution of the TCS via HGT helps microbial community to respond to niche-specific signals and ecogenetically adapt against stress [99].

### Metagenomic demarcations across pseudomonad gene complements

Global alignment based mapping of 5 metagenomic datasets [8] against *Pseudomonas* genomes (n = 18) revealed (Additional file 1: Table S1) maximum mapping for strain RL i.e. HCH dumpsite = 6,473, 1 Km = 3,855 and 5 Km = 2,161. However, for HCH contaminated pond soil metagenome, strain TKP showed maximum reads mapping (n = 3,44,108) followed by strain RL (n = 2,92,290). *P. aeruginosa* 213BR, 19BR along with rest of the *Pseudomonas* genomes used in this study showed negligible metagenomic mapping (Additional file 1: Table S1). On the other hand, metagenomic mapping of GSMs [55] against 18 reference genomes revealed maximum mapping for strain RL i.e. 1,425 reads from HCH contaminated pond metagenome dataset, which is absolutely pertinent because pond is the site of isolation of the strain RL. Our results validated GSM mapping to be more specific (species/strain) as compared to whole genome based metagenome mapping. Detailed results for metagenomic binning of reads for individual genome are provided in (Additional file 1: Table S1). NMDS ordination plots were used to determine the effect of HCH stress on the *in situ* abundance of these *Pseudomonas* cohorts (Figure 2) revealing strain TKP and RL to be the most enriched at the HCH dumpsite with maximum HCH contamination (450 mg HCH g^−1^) emphasizing the role of HCH stress in shaping up the genotypic heterogeneity.

Furthermore, pond sediment metagenome reads were mapped against *Pseudomonas* sp. RL genome to identify the environment specific metagenomic islands (MGIs). MGIs were then annotated (Additional file 3: Table S4) as continuous stretches of gaps in the metagenome recruitment plots. MGI annotations revealed that the metabolic functions such as divalent heavy metal cations, homoserine kinase, PIN domain of the 5′-3′ exonuclease, putative GTP-binding protein EngB, cytochrome, thiol-disulfide interchange protein, spermidine/putrescine ABC transporter ATP binding domain, are absent in the *in situ* cohorts of the strain RL. Detailed annotation results are provided in the supporting information i.e. Additional file 3: Table S4.

It has been reported that spermidine transporter demonstrates an important role in signal modulation of Type III secretory system [100]. Absence of both spermidine ABC transporter and type III secretory system in *Pseudomonas* sp. RL at the HCH contaminated pond clearly indicates their vestigiality under HCH stress. Similarly, GTP binding protein EngB is reported to be necessary for normal septation and its disruption leads to extensive filamentation. Interestingly, various pseudomonads e.g. *P. putida* and *P. aeruginosa*; are known to exert filamentous phenotype acquiring higher resistance towards various metabolic stress conditions [101]. As for an example, antibiotics stress has been reported to increase the filamentous growth in *P. aeruginosa* [102]. Our results here suggest that filamentous phenotype is favored across HCH stress. Molybdopterin co factor is required by enzymes for carbon, nitrogen and sulphur metabolism. However, its absence can be compensated by iron or other metal ions acting as cofactors via activating alternative nitrogenase or sulphur metabolism pathway. Besides above mentioned proteins, minimal genome at this site revealed the absence of flagellar basal body L-ring protein, flagellar rod assembly proteins, and flagellar basal body P-ring proteins (Additional file 3: Table S4). Interestingly, one of the MGIs was annotated to be N-acetyl neuramic acid synthetase (NcuB) which has been reported to be pathogenicity determinant in *Pseudomonas* species [103]. Our MGI analysis implies that virulence determinant factors such as NcuB, Type-III secretory system, spermidine transporter, and flagella-associated genes are inactive under HCH stress. This further highlights that virulence in *Pseudomonas* is a part of genetic adaptation course whereby it can reduce its virulence potential as a result of niche-specific selection [104]. Therefore, here we suggest that HCH stress might be causing the attenuation of virulence determining factors to acquire various habitat-specific metabolic traits. Although this is just a preliminary hypothesis and further research can yield valuable information on survival strategies of *Pseudomonas* at such stressed environment.

## Conclusions

Environment specific selective pressures have always been the apex driving force in the modulation of microbial genomes’ stability [105]. Genus *Pseudomonas* encompasses metabolically versatile and ecologically significant bacterial genotypes inhabiting all major natural environments [1]. Here, we have exploited the promising field of comparative genomics supplemented with community genomics to resolve the niche specific demarcations across 18 ecologically diverse *Pseudomonas* genotypes.

Various phylogenetic tools have been employed in the past for identification of *Bacillus*, *Clostridium* and *Pseudomonas* species based on 16S rRNA genes [106-108], However, here, we have used the conserved marker genes sequences (proteins = 400) supported by ANI for taxonomic assignment of strain RL. While 16S rRNA gene sequences are readily available, counting on one single gene lacks phylogenetic resolution [39]. Conserved gene based phylogenomic reconstruction revealed *P. aeruginosa* group to be phylogenetically as well as functionally conserved in contrast to *P. stutzeri* group which was found to deviate with ecotype status variations. Strain RL was found to deviate phylogenetically at species level from its reference genotypes (ANI value ≤ 80.03%). Further, while comparing the metabolic potential, strain-specific enrichment was discovered for α-linoleic acid degradation pathway and carbazole degradation in *Pseudomonas* sp. strain RL and *P. stutzeri* XLDN-R, respectively. We further reported (Figure 3) a mobile class-I integron (3,552 bp) in RL with intact 5’-CS (1,150 bp) and 3′-CS (1,200 bp) and *dhfrA1* (447 bp) as captured gene. Characterization of the integron further revealed presence of promoter sequence (Pc) upstream of the gene cassette highlighting its significance as an active natural expression vector. Metagenomic recruitment of the integron unit of RL on HCH contaminated metagenome datasets revealed (Figure 4) concentrated mapping for IS*6100* associated transposase-TnpA6100 gene, which indicated active transfer of DNA within or between genomes specifically at dumpsite (Figure 4). Higher abundance of integron mediated TnpA6100 can be attributed to the stress response by the bacterial community inhabiting the HCH contaminated sites, although the exact mechanism still remains unclear. Furthermore, MGIs were annotated to reveal vestigiality/loss of virulence determinants at the HCH dumpsite with respect to strain RL suggesting diminution of virulence factors at the expense of acquiring micro-habitat specific traits under stress.

### Availability of supporting data

The Whole Genome Shotgun project for *Pseudomonas* sp. strain RL genome sequence has been deposited at DDBJ/EMBL/GenBank under the accession JBOY00000000. The phylogenetic data for the study has been deposited in Dryad (http://datadryad.org/) with doi:10.5061/dryad.nd154.

## Additional files

Additional file 1: Table S1. Genome characteristics of 18 *Pseudomonas* genomes along with metagenomic recruitment data. Additional file 2: Figure S1. Schematic representation of integron associated elements as determined in reference genotypes of RL, i.e. *P. stutzeri* T13, *P. aeruginosa* 9BR, and *P. aeruginosa* 213BR. Percent identity and E-value for each element is with respect to RL, and is written in brackets below each segment. Horizontal arrows show gene orientation. Additional file 3:
**Table S2.** Table showing presence and comparison of response regulator proteins of Two-component system (TCS) in HCH-tolerant strains: RL, *P. aeruginosa* MTB1 and strain TKP. **Table S3.** A table showing potential HGT candidates in strain RL and its reference genotypes as predicted using SIGI-HMM. **Table S4.** Annotated MGIs in *Pseudomonas* sp. RL after the metagenomic recruitment of RL genome on pond metagenome reads. Additional file 4: Table S5. Table showing annotated orthologs between all 18 *Pseudomonas* strains. Additional file 5: Table S6. Table showing annotated orthologs between *P. stutzeri* and *P. aeruginosa.*
