# Supplementary material for: Pan-genome dynamics of Pseudomonas gene complements enriched across hexachlorocyclohexane dumpsite
Source: BMC Genomics. 2015 Apr 18;16(1):313. doi: 10.1186/s12864-015-1488-2 (PMC4405911; doi:10.1186/s12864-015-1488-2)
Supplement: Additional file 1: Table S1. — Genome characteristics of 18 Pseudomonas genomes along with metagenomic recruitment data. [file 12864_2015_1488_MOESM1_ESM.pdf]

Additional file 1

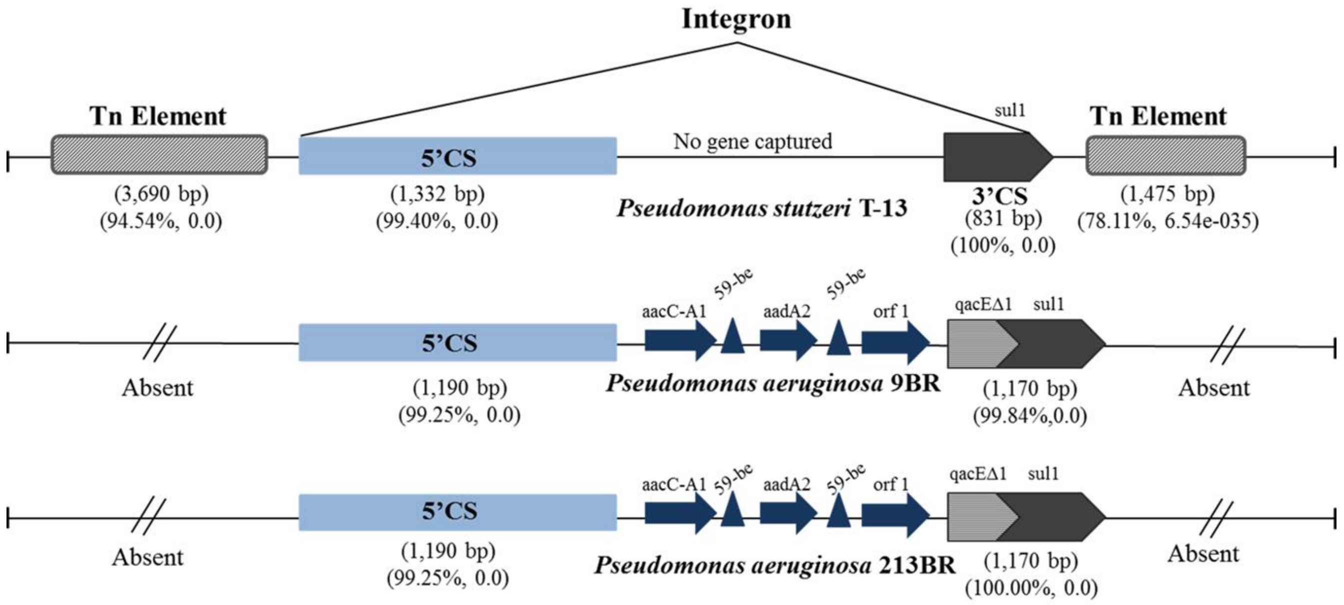

Figure S1: Schematic representation of integron associated elements as determined in reference genotypes of RL, i.e. *P. stutzer*i T-13, *P. aeruginosa* 9BR, and *P. aeruginosa* 213BR. Percent identity (BLASTX) and E-value for each element is with respect to RL, and is written in brackets below each segment. Horizontal arrows show gene orientation.
