# Supplementary material for: Pan-genome dynamics of Pseudomonas gene complements enriched across hexachlorocyclohexane dumpsite
Source: BMC Genomics. 2015 Apr 18;16(1):313. doi: 10.1186/s12864-015-1488-2 (PMC4405911; doi:10.1186/s12864-015-1488-2)
Supplement: Additional file 2: Figure S1. — Schematic representation of integron associated elements as determined in reference genotypes of RL, i.e. P. stutzeri T13, P. aeruginosa 9BR, and P. aeruginosa 213BR. Percent identity and E-value for each element is with respect to RL, and is written in brackets below each segment. Horizontal arrows show gene orientation. [file 12864_2015_1488_MOESM2_ESM.pdf]

Table S1: Genome characteristics of 18 *Pseudomonas* genomes along with metagenomic recruitment data.

Genome Features	1	2	3	4	5	6	7	8	9	10	11	12	13	14	15	16	17	18
**G+C Difference**	0.02	0.02	0.02	0.02	0.02	0.02	0.02	0.02	0.02	0.02	0.02	0.02	0.02	0.02	0.02	0.02	0.02	0.02
**Codon Bias**	0.11	0.13	0.12	0.12	0.12	0.11	0.15	0.14	0.13	0.13	0.14	0.09	0.12	0.10	0.11	0.10	0.12	0.1
**Genome size (In Mb)**	3.80	5.07	6.74	6.72	6.85	6.40	4.53	4.71	4.70	4.65	4.57	6.58	7.01	5.07	5.43	5.10	4.63	5.70
**Meta_1KM**	3,855	788	1,503	0	0	186	105	183	1,095	103	807	107	3,421	151	134	1	0	3
**Meta_5Km**	2,161	474	491	0	0	156	81	82	299	65	474	71	1,373	0	0	0	0	0
**Meta_DS**	6,473	3,415	3,101	11	1	1,006	455	1,006	2,133	604	2,613	358	6,389	1	121	12	14	53
**Meta_solexa_DS**	63	87,681	43,046	91	14	13,328	12,627	19,523	15,393	6,517	46,599	3,702	88,432	11	3	1	0	4
**Meta_Pond**	292,290	195,770	200,842	308	12	56,037	46,344	65148	57,526	26,055	192,949	22,054	3,44,108	156	178	0	1	3
**Pathways reconstructed**	89	97	101	101	101	97	99	86	92	91	90	99	93	69	69	72	67	77
**MGE**	24	42	30	27	28	36	25	41	39	12	70	8	56	3	2	21	82	26
**IS elements**	13	3	11	11	11	14	34	24	0	8	28	3	18	11	1	16	31	2

**GSMer 1km**	2	1	0	0	0	0	1	0	0	0	0	0	0	0	1	0	0	0
**GSMer 5km**	0	0	0	0	0	0	0	0	0	0	0	0	0	1	0	0	0	0
**GSMer DS**	0	3	0	0	0	0	0	2	0	1	0	0	0	3	0	0	0	0
**GSMer solexaDS**	230	917	25	28	19	2	297	181	305	67	87	5	0	0	0	0	0	0
**GSMer pond**	1425	2	3	1	1	0	5	14	16	20	4	0	0	0	0	0	0	0

Genomes are labeled as numbers from 1 to 18; numbers correspond to the serial number of genomes as they appear in Table 2 in the main article text.

Meta_1km, Meta_5km, Meta_DS, Meta_Pond, represents metagenomic reads recruitment from 1km, 5Km, Dumpsite and pond data on 1-13 genomes. Similarly, GSMer 1km, GSMer 5km, GSMer DS, GSMer pond, represents metagenomic recruitment of GSMs of 1-18 genomes.
