# Supplementary material for: Pan-genome dynamics of Pseudomonas gene complements enriched across hexachlorocyclohexane dumpsite
Source: BMC Genomics. 2015 Apr 18;16(1):313. doi: 10.1186/s12864-015-1488-2 (PMC4405911; doi:10.1186/s12864-015-1488-2)
Supplement: Additional file 3: — Table S2. Table showing presence and comparison of response regulator proteins of Two-component system (TCS) in HCH-tolerant strains: RL, P. aeruginosa MTB1 and strain TKP. Table S3. A table showing potential HGT candidates in strain RL and its reference genotypes as predicted using SIGI-HMM. Table S4. Annotated MGIs in Pseudomonas sp. RL after the metagenomic recruitment of RL genome on pond metagenome reads. [file 12864_2015_1488_MOESM3_ESM.pdf]

Table S2: Table showing presence and comparison of response regulator proteins of Two-component system (TCS) in HCH-tolerant strains: RL, *P. aeruginosa* MTB1 and strain TKP.

S.No.	Gene Name	Physiological Function	*Pseudomonas* sp. RL (%Identity, E-value)	*P. aeruginosa* MTB1 (%Identity, E-value)	*Pseudomonas* sp. TKP (%Identity, E-value)
**1.**	*cheR1*	Chemotaxis	79.60, 7.79e-078	99.29, 0.0	81.43, 4.70e-099
**2.**	*pilG*	Pilus production	88.77, 1.32e-117	99.76, 0.0	86.14, 2.71e-089
**3.**	*pilH*	Twitching Mobility	87.77, 6.30e-104	99.73, 0.0	85.48, 2.91e-067
**4.**	*pilI*	Pilus biosynthesis	84.04, 4.63e-084	100, 0.0	Absent
**5.**	*pilJ*	Pilus biosynthesis	88.74, 0.0	99.51, 0.0	81.23, 6.64e-177
**6.**	*algR*	Alginate biosynthesis	83.54, 5.06e-103	99.33, 0.0	Absent
**7.**	*cheB*	Chemotaxis	85.79, 4.68e-138	98.55, 0.0	84.42, 5.70e-105
**8.**	*cheY*	Chemotaxis	84.53, 4.51e-077	84.80, 3.18e-079	79.62, 1.19e-032
**9.**	*fleR*	Motility and adhesion	82.40, 1.44e-135	83.01, 1.057e-140	83.62, 0.0
**10.**	*gacA*	Quorum sensing and virulence factors	84.63, 1.84e-108	98.91, 0.0	79.14, 6.35e-048
**11.**	*narL*	Nitrate/nitrite respiration	79.62, 1.53e-019	Absent	81.56, 1.62e-085
**12.**	*phoB*	Phosphate regulation	87.41, 0.0	99.57, 0.0	86.81, 0.0

Table S3: A table showing annotated potential HGT candidates in strain RL and its reference genotypes as predicted using SIGI-HMM.

S.No	*Pseudomonas Genomes*	HGT candidates with assigned K0-Number using KAAS	Probable pathways mapped using MinPath
**1**	***P.* sp. RL**	Uncharacterized protein/K06959	RNA binding domain (ACLAME)
**2**	***P. stutzeri* A1501**	Uncharacterized protein/K06959	RNA binding domain (ACLAME)
**3**	***P. mendocina* ymp**	5-methyl tetrahydrofolate-homocysteine methyltransferase/K00548	1. Biosynthesis of amino acids<br>2. One carbon pool by folate<br>3. Biosynthesis of secondary metabolites<br>4. Selenocompound metabolism<br>5. Cysteine and methionine metabolism
**4**	***P. stutzeri* ATCC 14405**	nitrate reductase alpha subunit/K00370	1. Nitrogen metabolism<br>2. Two component system
**5**	***P. stutzeri* CCUG 29243**	5-methyl tetrahydrofolate-homocysteine methyltransferase/K00548	1. Biosynthesis of amino acids<br>2. One carbon pool by folate<br>3. Selenocompound metabolism<br>4. Cysteine and methionine metabolism
**6**	***P. stutzeri* XLDN-R**	nitrate reductase alpha subunit/K00370	1. Nitrogen metabolism<br>2. Two component system
**7**	***P. stutzeri* T13**	nitrate reductase alpha subunit/K00370	1. Nitrogen metabolism<br>2. Two component system
**8**	***P.* sp. TKP**	alcohol dehydrogenase (cytochrome c)/K00114	1. Glycolysis/Gluconeogenesis<br>2. Propanoate metabolism<br>3. Chloroalkane and chloroalkene degradation
**9**	***P. denitrificans ATCC 13867***	type I restriction enzyme, R subunit/KO 1153	NA

Table S4: Annotated MGIs in *Pseudomonas* sp. RL after the metagenomic recruitment of RL genome on pond metagenome reads.

S.No.	Coordinates (Begin)	Coordinates (End)	ORFs annotated	Function assigned
**1**	743459	753606	10	Ribonucleotide reductase, alpha subunit<br>Divalent heavy metal cations transporter<br>Homoserine kinase<br>Hypothetical protein<br>PIN domain of 5'-3' exonuclease<br>GTP binding protein-EngB<br>Cytochrome<br>Thiol-disulphide interchange protein<br>Exonuclease
**2**	757852	766941	1	Spermidine/putrescine ABC transporter
**3**	854524	856180	1	Molybdopterin converting factor, large and small subunits
**4**	862162	864142	1	ATP-dependent protease
**5**	879708	881968	1	ATP-dependent helicase HepA
**6**	897349	910283	2	Electron transport complex protein Rnfc<br>Electron tranposrt complex protein Rnfb
**7**	962380	964081	1	PhnA protein: part of a large operon associated with alkylphosphonate uptake and carbon-phosphorus bond cleavage
**8**	1422893	1406724	5	Unknown function protein DUF334<br>Unknown function protein DUF752<br>FAD dependent oxido reductase<br>Polyphosphate kinase<br>Nicotine nucleotide pyrophosphorylase
**9**	1422982	1434847	6	ABC type Fe3+ transport system<br>Periplasmic component<br>Peptidylprolyl isomerase FK3P type<br>Zn binding domain protein<br>C-terminal processing peptidase<br>Serine peptidase
**10**	1450029	1436815	4	Putative M18 family aminopeptidase 2<br>Adenyl cyclase toxin CyaB<br>Hypothetical protein<br>Cation/acetonide symporter

**11**	2196580	2189660	4	Hypothetical protein<br>Putative outer membrane transporter protein<br>Conserved hypothetical protein<br>Outer membrane heavy metal efflux protein-permease
**12**	2516904	2508048	2	Arabinose efflux permease family protein<br>Peptidylprolyl isomerase FK3P type
**13**	2521831	2536824		Glycogen synthase
**14**	2598875	2590005	2	N-actely neuramic acid synthetase NcuB<br>Thiamine pyrophosphate binding domain containing protein
**15**	2607521	2614867	3	Flagellar basal body L-ring protein<br>Flagellar basal body rod protein FlgG, FlgF<br>Flagellar basal body P-ring protein
**16**	2729503	2705723	4	Putrescine ABC transporter<br>Putative glutamine synthetase<br>Glutamate-ammonia ligase<br>Putative aminotransferase
**17**	3124352	3131784	5	Glutathione peroxidase<br>Acyl transferase<br>Peptidyl-prolyl cis-trans isomerase<br>Acetate kinase<br>Catalytic LigB subunit of aromatic ring opening-<br>dioxygenase
